# Glioblastoma mimicking a cerebral contusion: A case report

**DOI:** 10.3892/ol.2013.1537

**Published:** 2013-08-21

**Authors:** XINWEI LI, KUN WANG, ANLING ZHANG, ZHENGFEI SONG, SHUXU YANG, CONG QIAN, YIRONG WANG

**Affiliations:** 1 Department of Neurosurgery, Sir Run Run Shaw Hospital, Medical College, Zhejiang University, Hangzhou, Zhejiang 310016, P.R. China; 2 Department of Neurosurgery, Hangzhou Xiasha Hospital, Sir Run Run Shaw Hospital, Medical College, Zhejiang University, Hangzhou, Zhejiang 310016, P.R. China; 3 Department of Neurosurgery, Tianjin Medical University General Hospital, Tianjin, Hebei 300052, P.R. China; 4 Laboratory of Neurooncology, Tianjin Neurological Institute, Tianjin, Hebei 300052, P.R. China

**Keywords:** brain tumor, craniocerebral trauma, intratumoral hemorrhage, diagnosis, treatment

## Abstract

A 61-year-old male presented with a rare case of glioblastoma mimicking a cerebral contusion subsequent to collapsing. The patient had been medicated for hypertension for seven years and diabetes for eight years prior to hospitalization. Brain computed tomography (CT) revealed a cerebral contusion and intracerebral hemorrhage (ICH) in the left temporal region. The patient was initially administered intravenous drugs to reduce the intracranial pressure following the diagnosis of a cerebral contusion. Serial CT revealed ICH resorption. However, the patient was again admitted due to a headache and vomiting two months later. Magnetic resonance imaging (MRI) demonstrated an enhanced ring-shaped mass around the cyst cavity within the left temporal region, with surrounding edema. The patient underwent cyst puncture drainage in the temporal region. No tumor cells were identified in the cyst fluid and the culture was also negative. The patient was admitted for a headache and vomiting for the third time one month after being discharged. A cyst, tumor and meningoencephalitis were suspected following an MRI scan. The patient was treated with a left temporal craniotomy for a mass resection and biopsy. The histological diagnosis of the biopsy specimen was that of a glioblastoma. Two months later, MRI revealed a recurrence of the glioblastoma. In the present case, a brain tumor should have initially been suspected as the cause of the ICH, despite the history of craniocerebral trauma and hypertension. Early awareness of this potential cause of ICH may facilitate a more prompt diagnosis and treatment.

## Introduction

Highly-vascularized, malignant primary brain tumors (BTs), including glioblastoma, oligodendroglioma and metastatic BT, tend to bleed spontaneously and should always be included in the differential diagnosis of non-traumatic intracerebral hemorrhage (ICH) (1). However, cases of ICH that are caused by traumatic intratumoral hemorrhage (ITH) have rarely been reported (2,3). The incidence of ITH depends mainly on the tumor histology and location, which adversely affect the prognosis of the patients. The present study describes a case of glioblastoma mimicking a cerebral contusion, which resulted in a delay in the diagnosis and treatment of the patient. Written informed consent was obtained from the patient.

## Case report

**Figure 1:**
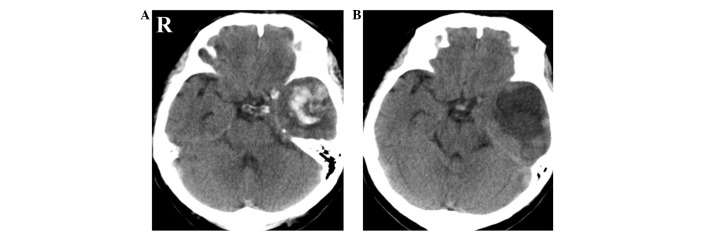
(A) Computed tomography (CT) showing a cerebral contusion and intracerebral hemorrhage (ICH) in the left temporal region. (B) CT showing ICH resorption.

**Figure 2:**
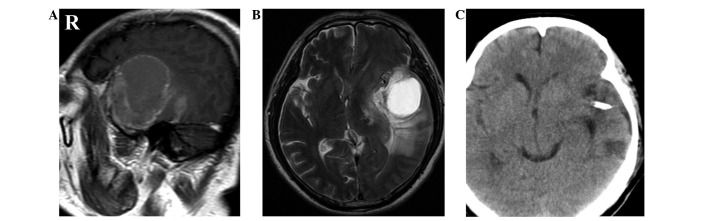
(A) T_1_-weighted magnetic resonance imaging (MRI) showing an enhanced ring-shaped mass around the cyst cavity within the left temporal region. (B) T_2_-weighted MRI showing a cyst cavity with surrounding edema. (C) Computed tomography (CT) showing cyst fluid resorption following puncture drainage.

**Figure 3:**
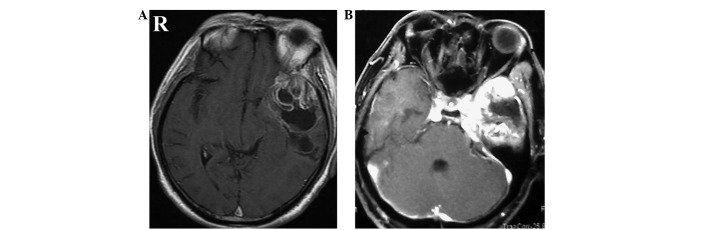
(A) Enhanced magnetic resonance imaging (MRI) showing an enhanced mass in the left temporal region. (B) Enhanced MRI showing the recurrence of an enhanced mass in the left temporal region.

A 61-year-old male, who had been medicated for hypertension for seven years and diabetes for eight years, was brought to the Emergency Department, Sir Run Run Shaw Hospital, Medical College, Zhejiang University (Hangzhou, China) subsequent to collapsing at home. The patient had exhibited the symptoms of a headache and vomiting for the last two days. The Glasgow Coma Scale score was E4V4M6 and the patient demonstrated mixed aphasia. Computed tomography (CT) revealed a cerebral contusion in the left temporal region (Fig. 1A). The patient was initially administered intravenous drugs to reduce the intracranial pressure following the diagnosis of a cerebral contusion and ICH. CT revealed ICH resorption a week later following the treatment (Fig. 1B). However, the patient was referred back to Sir Run Run Shaw Hospital due to a recurrent severe headache and vomiting two months after being discharged. Magnetic resonance imaging (MRI) revealed an enhanced ring-shaped mass around the cyst cavity, with surrounding edema in the left temporal region (Fig. 2A and B). Under local anesthesia, the patient was treated by cyst puncture drainage within the temporal region, following which, a steady recovery was made. CT revealed cyst fluid resorption (Fig. 2C). The histopathological result did not show any tumor cells in the cyst fluid and the culture was also negative. The patient was readmitted for the third time with the same recurrent symptoms. A cyst, tumor and meningoencephalitis were suspected following enhanced MRI (Fig. 3A). The patient underwent a left temporal craniotomy for a mass resection and biopsy under general anesthesia. The histological diagnosis was that of glioblastoma multiforme. Two months later, MRI revealed a recurrence of the glioblastoma (Fig. 3B). The patient received no further treatment and succumbed to the disease.

## Discussion

ICH accounts for nearly one-half of all cerebrovascular events (4). Clinical and autopsy studies have identified that BTs represent 0.9–11% of spontaneous ICH (5–14). However, cases of ITH caused by craniocerebral trauma have rarely been reported (2,3). It has been well established that the structure and function of blood vessels becomes markedly abnormal in brain tumors (15). Goetting and Swanson (16) suggested that an abrupt, large increase in arteriovenous pressure creates a transient gradient between the vascular and extravascular intracranial compartments, which may lead to the rupture of fragile tumor vessels.

The approach to the present case had been biased towards the treatment of a cerebral contusion in the left temporal region due to the initial pretreatment CT and pre-existing hypertension. The diagnosis of a cerebral contusion and ICH was easily justifiable, which attributed to the delayed correct diagnosis and treatment of the patient. Initially, only CT was performed, as craniocerebral trauma was the only cause of the ICH and also as the conservative treatment was apparently effective. Cyst puncture drainage was performed in the temporal region and, notably, the histological examination revealed no tumor cells in the cyst fluid and the cyst fluid culture demonstrated negative results. These results confirmed the first diagnosis until the pathological investigation finally revealed the definitive diagnosis of a glioblastoma.

The present case shows that pre-operative CT with or without MRI does not exclude brain tumors as the cause of ICH, despite the present patient having a history of craniocerebral trauma and hypertension. Up to 10% of patients with BTs may experience a diagnostic delay if CT is the only imaging modality that is used (17). Dual-energy CT may be useful in detecting underlying tumors in patients with an ICH of unknown origin, and is a useful tool in differentiating between tumor bleeding and pure ICH in patients with acute ICH of an unclear origin (18). Using MRI with gadolinium early in the post-operative period is likely to lead to an earlier detection of the BT. A previous study showed that the use of MR angiography (MRA) aided the disclosure of the development of an intratumoral aneurysm on a dilated feeding artery, the rupture of which led to intratumoral bleeding (19). The standard treatment of a BT manifesting as an ICH is the surgical removal of the hematoma and the tumor (13,20). However, the optimal timing of the therapeutic intervention is poorly defined, particularly when the neurological status of the patient is stable following admission and there is only a minimal or no mass effect on the CT scan (21).

In the present case, since the signs and symptoms developed shortly following a head trauma, it is possible that the trauma participated in the induction of the hemorrhage. The numerous tumor vessels and microcysts may have been distorted by the force of the impact, causing their thin walls to rupture. In such cases, BT should be suspected as a cause of ICH, despite a history of craniocerebral trauma and hypertension. An appropriate and prompt investigation should be performed and clinical follow up is also essential for detecting subtle neurological deterioration of the patient to avoid a delay in the diagnosis and treatment.
